# Dietary aquaculture by-product hydrolysates: impact on the transcriptomic response of the intestinal mucosa of European seabass (*Dicentrarchus labrax*) fed low fish meal diets

**DOI:** 10.1186/s12864-018-4780-0

**Published:** 2018-05-24

**Authors:** Alexandre Leduc, Céline Zatylny-Gaudin, Marie Robert, Erwan Corre, Gildas Le Corguille, Hélène Castel, Antoine Lefevre-Scelles, Vincent Fournier, Enric Gisbert, Karl B. Andree, Joël Henry

**Affiliations:** 1 Normandie University, UNICAEN, Sorbonne Universités, MNHN, UPMC Univ Paris 06, UA, CNRS, IRD, Biologie des Organismes et Ecosystèmes Aquatiques (BOREA), Université de Caen Normandie, Caen, 14032 France; 2 UPMC, CNRS, FR2424, ABiMS, Station Biologique, Roscoff, France; 3 Normandie University, UNIROUEN, INSERM, U1239, Laboratoire Différenciation et Communication Neuronale et Neuroendocrine, Institut de Recherche et d’Innovation Biomédicale de Normandie, Rouen, France; 4 Aquativ (DianaAqua, Symrise Group), Elven, France; 5 Institute of Agriculture and Food Research and Technology (IRTA), Sant Carles de la Ràpita, Spain

**Keywords:** Hydrolysate, Fishmeal replacement, By-products, Aquaculture, Aquafeed, European seabass, Illumina RNA-sequencing, Intestinal organization, Metabolic pathways

## Abstract

**Background:**

Aquaculture production is expected to double by 2030, and demands for aquafeeds and raw materials are expected to increase accordingly. Sustainable growth of aquaculture will require the development of highly nutritive and functional raw materials to efficiently replace fish meal. Enzymatic hydrolysis of marine and aquaculture raw materials could bring new functionalities to finished products. The aim of this study was to determine the zootechnical and transcriptomic performances of protein hydrolysates of different origins (tilapia, shrimp, and a combination of the two) in European seabass (*Dicentrarchux labrax*) fed a low fish meal diet (5%), for 65 days.

**Results:**

Results were compared to a positive control fed with 20% of fish meal. Growth performances, anterior intestine histological organization and transcriptomic responses were monitored and analyzed. Dietary inclusion of protein hydrolysates in the low fish meal diet restored similar growth performances to those of the positive control. Inclusion of dietary shrimp hydrolysate resulted in larger villi and more goblet cells, even better than the positive control. Transcriptomic analysis of the anterior intestine showed that dietary hydrolysate inclusion restored a pattern of intestinal gene expression very close to the pattern of the positive control. However, as compared to the low fish meal diet and depending on their origin, the different hydrolysates did not modulate metabolic pathways in the same way. Dietary shrimp hydrolysate inclusion modulated more metabolic pathways related to immunity, while nutritional metabolism was more impacted by dietary tilapia hydrolysate. Interestingly, the combination of the two hydrolysates enhanced the benefits of hydrolysate inclusion in diets: more genes and metabolic pathways were regulated by the combined hydrolysates than by each hydrolysate tested independently.

**Conclusions:**

Protein hydrolysates manufactured from aquaculture by-products are promising candidates to help replace fish meal in aquaculture feeds without disrupting animal metabolism and performances.

**Electronic supplementary material:**

The online version of this article (10.1186/s12864-018-4780-0) contains supplementary material, which is available to authorized users.

## Background

Fish captures have been stabilizing since the 1980’s, and in most parts of the world seafood production is now supplied by the aquaculture industry [1]. Aquaculture production is expected to double by 2030 to meet customer demand, so demands for aquafeeds and raw materials will increase accordingly [2]. Originally, fish meals (FM) manufactured from wild fish were primarily used for growing carnivorous fish species. This kind of raw material is an excellent source of highly digestible protein and fat, is well balanced in essential amino acids, and has good palatability properties [3]. However, FM availability from wild fish is limited, its price is volatile, and its inclusion at high levels in aquafeeds is not sustainable; according to Duarte et al., FM and fish oil will be depleted by 2040 [4]. Consequently, sustainable growth of aquaculture will require the development of highly nutritive and functional raw materials to replace FM efficiently. Many studies have been conducted on carnivorous species to evaluate the performance of plant-based meal (PBM) diets to efficiently replace dietary FM; many of them were remarkably efficient, with partial or total substitution of FM by PBM [5–8]. However, replacing FM by high levels of PBM may also reduce feed palatability and fish growth [9, 10]. Imbalanced amino acid composition of PBM leads to nutritional deficiency, and feed formulae incorporating high levels of such raw materials have to be supplemented in essential free amino acids [11]. It is also now well-documented that plants contain endogenous anti-nutritive compounds and complex carbohydrates that could affect nutrient digestibility and thereby negatively impact nutritional performances [12, 13]. Moreover, high dietary inclusion of PBM in feeds for carnivorous species leads to enteritis [14, 15], as well as depressed immunity [16, 17]. Trials had been conducted about FM replacement by PBM on European seabass and it had been shown in different trials that it was possible to replace up to 50% of dietary FM by plant based meal without modifying fish growth performances (for review Kousoulaki et al., 2015) [18].

The large availability of marine and aquaculture by-products could allow for partial replacement of wild fish-derived FM [19, 20]. By-products from the processing of fishery and aquaculture animals are more and more considered as a potential source of raw materials for sustainable FM production [21–23]. However, the quality of the processes applied to raw materials is critical to enhance their nutritional value. In this sense, enzymatic hydrolysis could improve the palatability, nutritional quality and functional properties of the finished product [24–26]. Enzymatic hydrolysis of fish proteins results in the formation of a mixture of free amino acids, di-, tri- and oligo-peptides, and enhances the occurrence of polar groups and the solubility of hydrolysate compounds [27]. Because low-molecular-weight nitrogenous compounds are important for the feeding behavior [28], nutrition [29–31] and health [32, 33] of aquaculture species, protein hydrolysates could be good candidates for high FM substitution in aquafeeds. However, protein hydrolysate performances could be highly dependent on the methods used for their production: their nutritional and functional properties are closely related to their characteristics and composition, including the abundance and diversity of different oligo-peptides [34, 35].

Dietary shrimp hydrolysates stimulate growth performances in fish, and also possess antimicrobial properties against aquaculture pathogens [36–39]. Similarly, tilapia hydrolysates as well as other fish hydrolysates have been evaluated; functional properties have been evidenced, such as antioxidant [24] and antimicrobial [40] activities. In particular, Khosravi et al. showed that high levels of FM could be replaced by low levels of protein hydrolysate combined with PBM in aquafeeds [38]. In addition, health benefits (i.e. immunity and gut cellular organization) of dietary protein hydrolysates have been reported in different fish species under challenging conditions [32, 38, 39, 41].

The European seabass (*Dicentrarchus labrax*, Linnaeus, 1758) is a marine fish widely reared in the Mediterranean sea, with more than 156,449 tons produced in 2014 [1]. It is a strictly carnivorous species [42] that requires a high level of animal proteins in its diet. For instance, the natural diet of wild European seabass contains ca. 43–50% of animal proteins [43], whereas aquafeeds for this species generally contain at least 20% of FM to support good fish performances [44]. Thus, replacing FM in European seabass aquafeed is still a major objective for the aquaculture industry. Although the complete replacement of FM by PBM has been reported as possible for European seabass, feed formulation required palatability enhancers to improve feed intake, as well as amino acid supplementation to avoid nutritional deficiency linked to PBM [6]. Trials recently conducted in European seabass showed that 5% inclusion of shrimp- and tilapia-based protein hydrolysates associated with a combination of PBM successfully replaced 15% FM without affecting growth or health performances [45].

The intestine is involved not only in digestion and feed absorption, but also in water and electrolyte balance, nutrient sensing, and immunity. This functional diversity is gradually being elucidated in fish, as different histological and molecular approaches provide new items of knowledge regarding the many vital functions conducted along the gastrointestinal tract [46, 47]. In this view, the numbers of transcriptomic studies in aquaculture have increased, mostly in the field of nutrition and immunity. Many of them have focused on understanding how specific diets and functional ingredients could modulate metabolic pathways and regulate specific tissue expression [48–50]. But the effect of dietary protein hydrolysates on the regulation of fish metabolism has never been investigated so far.

We evaluated the effects of protein hydrolysates of different origins on European seabass fed a low FM diet. In addition to growth performances, we also studied the cellular organization and gene responses of the intestinal mucosa to investigate the effects of dietary protein hydrolysates on fish metabolism.

## Methods

### Diets

Five diets were formulated as follows: 2 diets containing FM at 5 and 20% of dry matter (diets FM5 and FM20) and 3 more diets containing 3 protein hydrolysates, shrimp-based hydrolysate (SH), tilapia hydrolysate (TH) and a 50/50 mixture of the two (MH), included at 5% of dry matter in the FM5 diet (Additional file 1). Both protein hydrolysates were provided by Aquativ (Diana Aqua, Symrise group, Elven, France). They were produced from the cephalothorax of white shrimp (*Litopenaeus vannamei*) and from Nile tilapia (*Oreochromis niloticus*) carcasses obtained from commercial food processing plants. These two protein hydrolysates have very different peptide profiles [37, 40]. All the diets were balanced for deficient amino acids according to the requirements determined for European seabass [51]. Diets manufactured by BIOMAR (Tech Centre, Brande, Denmark) were extruded with 2 different pellet diameters – 1.5 and 2.5 mm – for them to be adapted to the size of the fish during the trial. Diets were isoproteic (42.7 ± 1.1% of crude protein), isolipidic (19.3 ± 0.5% of crude fat) and isoenergetic (5.2 ± 0.1 kJ/kg).

### Animals and feeding trial

The feeding trial was conducted at the Institut de Recerca i Tecnologia Agroalimentàries (IRTA, Sant Carles de la Rapita, Spain). European seabass (body weight, BW = 2.0 ± 0.2 g) were obtained from Piscicultura Marina Mediterránea SL (Andromeda Group, Burriana, Valencia, Spain). They were fed a commercial feed (OptibassL-2, Skretting, Spain; 48.5% of proteins, 16% of lipids, 3.7% of fibers, 6.4% of ashes) for 2 weeks for them to acclimate to the experimental facilities. Then they were randomly distributed into twenty 500-L fiberglass circular tanks (5 diets, 4 replicates *per* diet) at an initial density of 0.4 kg/m^3^ (100 fish *per* tank). Before the feeding trial, sea bass were individually weighed for BW and measured for standard length (SL) (BW = 2.2 ± 0.01 g; SL = 5.1 ± 0.04 cm). During the study, average water temperature and pH (pH meter 507; Crison Instruments), salinity (MASTER-20 T; ATAGO Co. Ltd) and dissolved O_2_ (OXI330; Crison Instruments) were 23.2 ± 0.5 °C, 7.5 ± 0.2, 35.8 ± 0.3 ppm and 6.2 ± 1.2 mg/L, respectively. The water flow rate in the experimental tanks was maintained at approximately 9.0 L/min by means of a recirculation system (IRTAmar®) that maintained adequate water quality (ammonia: 0.08 ± 0.04 ppm, nitrites: 0.032 ± 0.02 ppm) through UV, biological and mechanical filtration. The photoperiod followed seasonal changes (February–April; latitude 40°37’41’N). Sea bass were fed in excess 6 times a day with automatic feeders (ARVO-TEC T Drum 2000™, Arvotec, Huutokoski, Finland) at a ration rate of 4.5%/day for 65 days. On day 31, the BW of 50 fish *per* tank was recorded to adjust the daily feed ration.

At the end of the trial, we measured the BW and SL of all sea bass from each tank (the fish were fasted for 24 h prior to sampling). Specific Growth Rates (SGR), Fulton’s condition factors (K), feed conversion ratio (FCR) and Survival Rates (SR) were calculated as follows: SGR (% BW/day) = 100 x [ln final BW - ln initial BW]/duration of the trial (days); K = [BW/SL^3^] × 100; FCR = [kg diet consumed]/[kg final biomass − kg initial biomass + kg sampled fish + mortalities]; SR (%) = [number of fish at the end of the trial/initial number of fish] × 100.

### Ethics statement

All experimental procedures involving sea bass were conducted in compliance with the experimental research protocol approved by the Committee of Ethics and Animal Experimentation of the IRTA, the Departament Agricultura, Ramaderia, Pesca, Alimentació i Medi Natural (permit number 7962) and in accordance with the Guidelines of the European Union Council (86/609/EU) for the use of laboratory animals.

### Sample collection

During handling and weighing, sea bass were anesthetized with 50 mg/L of MS-222 (Sigma Aldrich, Saint-Louis, MO, USA). At the end of the trial, they were sacrificed with an overdose of MS-222 (100 mg/mL) to collect the different tissues for analytical purposes. Sea bass were sampled on a cold plate (0–4 °C), and the intestines from 15 individuals *per* diet were dissected and preserved in a 10% phosphate-formaldehyde buffer (pH = 7.2) for histological purposes. In addition, the anterior intestine from nine fish *per* dietary treatment was sampled and immediately flash-frozen in liquid nitrogen for transcriptomic analysis. These samples were kept at − 80 °C until RNA extraction.

### Histological organization of the intestine

After fixation, samples were dehydrated in a graded series of ethanol, cleared with xylene, embedded in paraffin (Histolab ZX-60 Myr, Medical Specialties MYR SL, Tarragona, Spain) and cut into serial sections (2–3 μm thick) (HM Microm, Leica Microsystems, Nussloch, Germany). Sections were stained with Hematoxin-Eosin for general histological descriptions, whereas slides were stained with Periodic Acid Schiff (PAS) and Alcian blue (AB) at two different pH values (1.0 and 2.5) to stain different types of mucins produced by goblet cells [52]. PAS stains neutral mucins produced by intestinal goblet cells in magenta, whereas AB weakly stains ionised sulphated glycoconjugates at pH = 1.0 and sialic acid at pH = 2.5. All sections were observed under a light microscope (Leica DM LB; Leica Microsystems) and photographed (Olympus DP70 Digital Camera; Olympus Imaging Europa GmbH, Hamburg, Germany). Digital images were processed and analyzed using ANALYSIS image analysis software package (Soft Imaging Systems GmbH). Total numbers of goblet cells (full and empty) and villi height were measured based on the analysis of eight to ten randomly chosen fields from the intestinal mucosa of 15 sea bass *per* diet. Goblet cell counts in intestinal villi were expressed over a contour length of 100 μm, whereas villi height and width were calculated according to the method of Escaffre et al. [53].

### Illumina sequencing

Total RNAs from a pool of the proximal intestines of three sea bass from the same replicate tank were extracted separately. Tissues were homogenized (Mini-Beadbeater, Biospec Products Inc., USA) in 1 mL of TRIzol (Ambion, Life Technologies, Carlsbad, CA, USA), and solvent extraction was performed following the manufacturer’s instructions. Final RNA concentrations were determined by spectrophotometry (NanoDrop 2000; Thermo Fisher Scientific, Waltham, Massachusetts, USA). RNA quality was assessed from A_260_/A_280_ ratios, and RNA integrity was assessed by denaturing gel electrophoresis. The three RNA samples from same replicate tank were then pooled before generating cDNA libraries. The total RNA concentration of each sample was quantified using a NanoDrop spectrophotometer (Thermo Fisher Scientific). cDNA libraries were built, and sequencing was performed as described in [54], with slight modifications. One μg of total RNA from each sample was initially used. dsDNAs were cleaved into 300-bp fragments using a Covaris S220 sonicator (Covaris Inc., Woburn, MA, USA) (duty cycle: 5%, intensity 3, 200 bursts *per* second, duration: 50 s). Eight pM of cDNA libraries *per* lane were loaded onto flow cells (Illumina Inc., San Diego, California, USA). The sequencing of 100-pb paired-end reads was performed on an Illumina Miseq Sequencer at the SéSAME Platform (Centre de Lutte Contre le Cancer François Baclesse, Caen, France).

### Bioinformatic analysis

Bioinformatic analysis was performed on RNA sequencing data from European seabass anterior intestine to study their physiological and molecular responses to dietary hydrolysate inclusion in a low fish meal diet. We tested 5 dietary conditions on the transcriptomic response of sea bass intestine: FM5 and FM20 diets as negative and positive control groups, respectively, and the SH, TH and MH hydrolysate-based diets as experimental groups. The whole raw dataset was filtered and trimmed using Trimmomatic v(0.30) [55], using the following parameters: ILLUMINACLIP:adapter.fa:2:30:10, LEADING:5, TRAILING:5, SLIDING WINDOW:4:5, MINLEN:25. Global sequence quality was checked using FastQC (v 0.11.3) (https://www.bioinformatics.babraham.ac.uk/projects/fastqc). Global assembly was conducted using Trinity 2.1.1. [56], a dedicated package for de novo transcriptomics. A normalization step was conducted according to kmer coverage (kmer of 25 nt, maximum coverage of 30) proposed by the Trinity package. The inconsistent contigs generated by Trinity were removed after a remapping of reads using Bowtie (v 1.1.2) [57], and estimating relative abundance using RSEM [58] to get the FPKM values (v 1.2.22) (the two software programs were launched through perl wrappers provided by the Trinity package). Finally, only transcripts with at least an FPKM value above 1 and isoforms corresponding to more than 1% of the total gene count were kept. Annotation of contig sequences was performed using both Blast2Go software [59] and the Trinotate pipeline (http://trinotate.github.io), as described in [60]. Sequences were blasted against the NCBI nr database (release 193) with the following set-up parameters: max BLAST hits 20, min Expect Value 10^− 3^, and against the human proteome Ensembl database (release 82) (BLASTX). Only hits with E-values < 0.001 were kept. Peptide prediction was performed using Transdecoder [60]. Similarity search (blastp of the Transdecoder-predicted peptides) was performed against the uniprot-swissprot database (release 2015–05). Peptide signal prediction was performed using signalP v4.1 [61]. Transmembrane peptide detection was performed using TMHMM v2.0c [62]. Protein domain search was performed using hmmscan from the hmmer v.3.1b1 suite against the Pfam-A database [63]. Finally, transcriptome functional annotation was performed using the Trinotate pipeline. A Gene Ontology (GO) classification was assigned to each predicted protein in BLASTX (E-Value hit: 10^− 6^, annotation cut-off: 55, GO weight: 5). Kyoto Encyclopedia of Genes and Genomes (KEGG) annotation was based on best BLASTX and PFAM search results.

### Differential expression analysis

Differential gene expression between dietary treatments was identified using R software [64] and the DESeq2 package [65]. Multiple testing was accounted for by controlling the false discovery rate (FDR) at 5% using the Benjamini-Hochberg procedure. The RNA targets with adjusted *P*-values < 0.2 and absolute fold changes ≥1.4 were considered as differentially expressed. The relatively low stringency of the cut-off criteria is consistent with other nutrigenomic studies [48]. The impacts of hydrolysate diets on specific pathways were monitored using Ingenuity Pathway Analysis software (Qiagen, Hilden, Germany) with standard parameters. All annotated regulated genes were used.

### Statistics

Final BW, SL and K results were expressed as mean ± standard error of the mean (SEM) from all fish from each treatment. SGR, intestinal villi height, number of goblet cells, proximal composition and survival rates were expressed as mean ± standard error of the mean (SEM) from the value of each replicate tank. All data were analyzed by one-way analysis of variance (ANOVA) followed by Tukey’s test (normality and homogeneity of variances were previously checked). Differences were considered significant at *P* < 0.05. For GO term comparison with Wego tool, Pearson Chi-Square tests were performed to show significant differences between diets (*P* < 0.05) [66].

## Results

### Zootechnical performances

Sea bass and feed performances are summarized in Table 1. At the end of the trial, dietary FM reduction (FM5 diet) had significantly impaired growth performances as compared to the positive control group (FM20) (*P* < 0.05). However, diets including protein hydrolysates (FM5 +  5% TH, FM5 +  5% SH and FM5 + 5% MH) significantly improved growth performances as compared to FM5 (*P* < 0.05) and allowed for similar growth performances as in sea bass fed the FM20 diet. The condition factor (K) and the feed intake were not affected by dietary FM reduction or dietary hydrolysate inclusion (*P* > 0.05). Even if FCR values were slightly improved in fish fed dietary hydrolysates compared to negative control (FM5), the only statistical difference was between the negative and the positive controls (*P* < 0.05). Dietary treatments did not impact fish survival (*P* > 0.05).

**Table 1:** Growth performances and survival rates of sea bass fed experimental diets over a 10-week period

DIET	FM5	FM20	FM5 + 5% TH	FM5 + 5% SH	FM5 + 5% MH
Initial BW (g)	2.2 ± 0.01	2.2 ± 0.01	2.2 ± 0 .01	2.3 ± 0.00	2.2 ± 0.01
Final BW (g)	11.7 ± 0.4^a^	13.1 ± 0.3^b^	13.9 ± 0.4^b^	13.2 ± 0.5^b^	13.0 ± 0.6^b^
SGR (%)	2.5 ± 0.1^a^	2.7 ± 0.1^b^	2.8 ± 0.1^b^	2.7 ± 0.1^b^	2.7 ± 0.1^b^
K factor	2.0 ± 0.03	2.0 ± 0.01	2.0 ± 0.02	2.0 ± 0.02	2.0 ± 0.03
Feed intake (g/kg ABW/d)	33.34 ± 1.46	30.40 ± 1.63	31.55 ± 1.41	30.60 ± 1.12	31.50 ± 0.94
FCR	1.95 ± 0.04^a^	1.75 ± 0.05^b^	1.83 ± 0.04^ab^	1.79 ± 0.04^ab^	1.82 ± 0.04^ab^
SR (%)	93.0 ± 2.6	96.0 ± 0.9	96.0 ± 0.9	93.8 ± 0.8	97.0 ± 1.1

Values are the means of four replicate groups of 50 measurements each, presented as mean ± SEM. Lines with different superscript letters differ significantly according to Tukey’s multiple comparison test (*P* < 0.05). Abbreviations: *FM5* 5% fish meal diet, *FM20* 20% fish meal diet; *TH* tilapia hydrolysate diet, *SH* shrimp hydrolysate diet, *MH* mixed hydrolysate diet, *BW* body weight, *SGR* specific growth rate, *ABW* average body weight, *FCR* feed conversion ratio, *SR* survival rate

### Histological examination

**Fig. 1:**
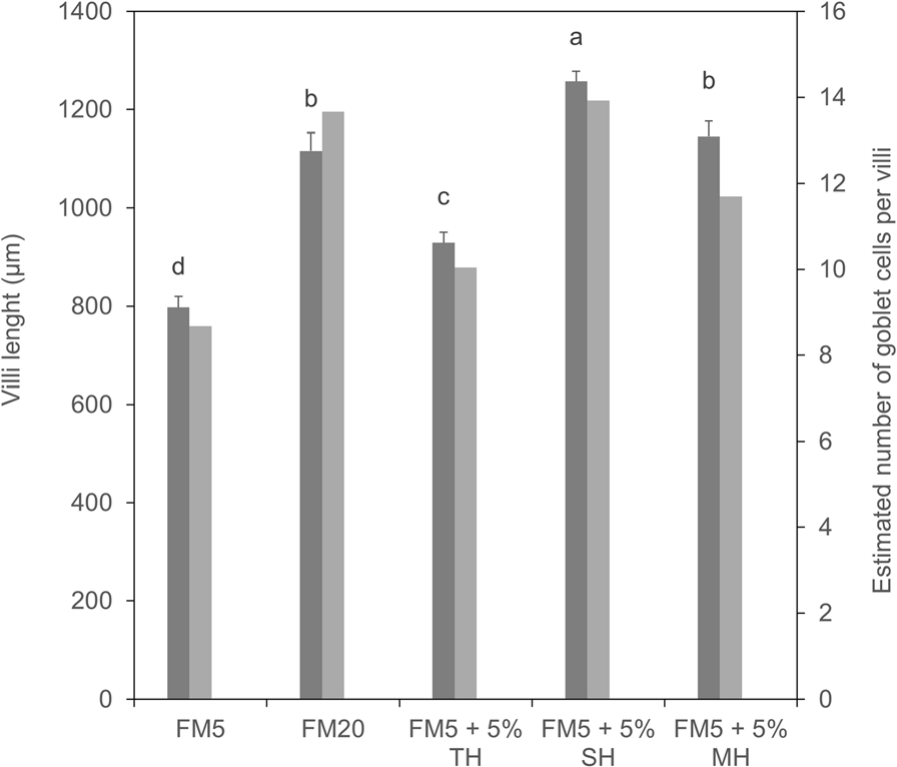
Villi height and goblet cell density. Villi height values (dark grey) are the means of four replicate groups of 15 measurements each, presented as mean ± SEM. Histograms with the same superscript letter did not differ significantly (Tukey’s multiple comparison test, *P* > 0.05). The number of goblet cells within 100 μm of intestinal epithelium (grey) was calculated from 15 individuals *per* dietary group. FM5, 5% fish meal diet; FM20, 20% fish meal diet; TH, tilapia hydrolysate diet; SH, shrimp hydrolysate diet; MH, mixed hydrolysate diet

Figure 1 shows the mean intestinal villi height of European seabass fed each diet. A significant decrease of villi height was observed in sea bass fed the FM5 diet as compared to the FM20 diet (*P* < 0.05). Inclusion of protein hydrolysates in the FM5 diet significantly increased intestinal villi height to reach values close to or even higher than in sea bass fed the FM20 diet (*P* < 0.05). The response of intestinal globlet cell density to dietary FM reduction and protein hydrolysate supplementation showed the same trend as intestinal villi height.

### Global overview of the RNA-seq

The whole European seabass transcriptome project includes 108 Illumina libraries corresponding to the anterior intestine, but also the liver and kidney for each diet, for a total of 832,132,824 paired-end reads of 100 bp. Table 2 presents an overview of the sequencing project. Mapping was conducted both on the reference seabass genome (http://seabass.mpipz.de/DOWNLOADS/) and on the de novo assembled transcriptome from all samples, corresponding to 36 experimental conditions (6 diets * 3 tissues * 2 fish conditions) realized in triplicate.

**Table 2:** Overview of sequencing, assembly and annotation

Metrix	Unfiltered	Filtered
Transcripts	625,845	56,246
Genes	467,824	39,180
GC%	44.28	44.13
Median length (bp)	389	910
Average length (bp)	922.7	1430.28
Min (bp)	201	201
Max (bp)	58,543	24,803
Total number of bases	577,467,800	80,447,300
Annotation		
Blastx hits with uniprot	278,406	45,853
Blastx hits with uniref90	377,852	46,552
Blastp hits with uniprot	227,499	41,578
Blastp hits with uniref90	280,111	42,257
Proteins with signal peptides	20,540	2226
Proteins with transmembrane helices	28,482	5034
Proteins with PFAM domains	216,290	32,907
Proteins with GO terms	71,304	45,454
Proteins with KEGG	181,895	41,993

Transcript filter: < 1 FPKM, isoform < 1%

Mapping scores were as follows: 83.55% for the mRNA extracted from the reference seabass genome annotation, and 92.77% for the de novo assembled transcriptome of the anterior intestine. In addition, the numbers of DE genes and annotations were higher with the de novo assembled transcriptome of the anterior intestine. Further differential expression analyses were then conducted on the global de novo transcriptome, composed of 56,246 transcripts after filtering with FPKM < 1 and isoform < 1%. The transcriptome corresponding to the total expression in the anterior intestinal mucosa was composed of 34,174 transcripts. More precisely, 99% of total expression was present in 49,797 for the whole project and in 10,003 transcripts for the anterior intestinal mucosa respectively.

### Quantitative analysis of differential gene expression

Differential gene expression analysis of RNA-seq data was performed to compare diets containing hydrolysates included to low (FM5) and high (FM20) fish meal diets. A total of 383 unique genes were differentially expressed (Table 3). Compared to the FM5 diet, 46 and 50 genes were differentially regulated by diets containing TH and SH, respectively, and this number was higher in the FM20 (197) and MH (270) groups. Compared to the FM5 diet again, the distribution of differentially regulated genes between the FM20 diet and diets including hydrolysates indicated that 30.4% (14/46), 66.0% (33/50), and 39.3% (106/270) of regulated genes were shared with the TH, SH and MH diets, respectively. A total of 301 genes were only regulated by diets containing hydrolysates, but not by the FM20 diet, whereas some genes were regulated only by a specific hydrolysate diet. In particular, 19, 8, and 222 genes were specifically regulated by the TH, SH and MH diets, respectively. Finally, the TH, SH and MH diets induced gene expression patterns very similar to the FM20 diet pattern, with only 8, 6, and 6 genes differentially expressed among these dietary groups, respectively. The complete list and corresponding fold changes of regulated genes is provided in Additional files 2 and 3, for a comparison with the low-FM (FM5) and high-FM (FM20) diets, respectively.

**Table 3:** Number of differently regulated genes as compared to the low (FM5) and high (FM20) fish meal diets

Nb of regulated genes	FM5	FM20	FM5 + 5% SH	FM5 + 5% TH	FM5+ 5% MH
vs. FM5	–	197	50	46	270
Shared with FM20		197	33	14	106
Specifically regulated	–	82	8	19	222
vs. FM20	197	–	8	6	6

*FM5* 5% fish meal diet, *FM20* 20% fish meal diet, *TH* tilapia hydrolysate, *SH* shrimp hydrolysate, *MH* mixed hydrolysate

### Identification of regulated genes

**Fig. 2:**
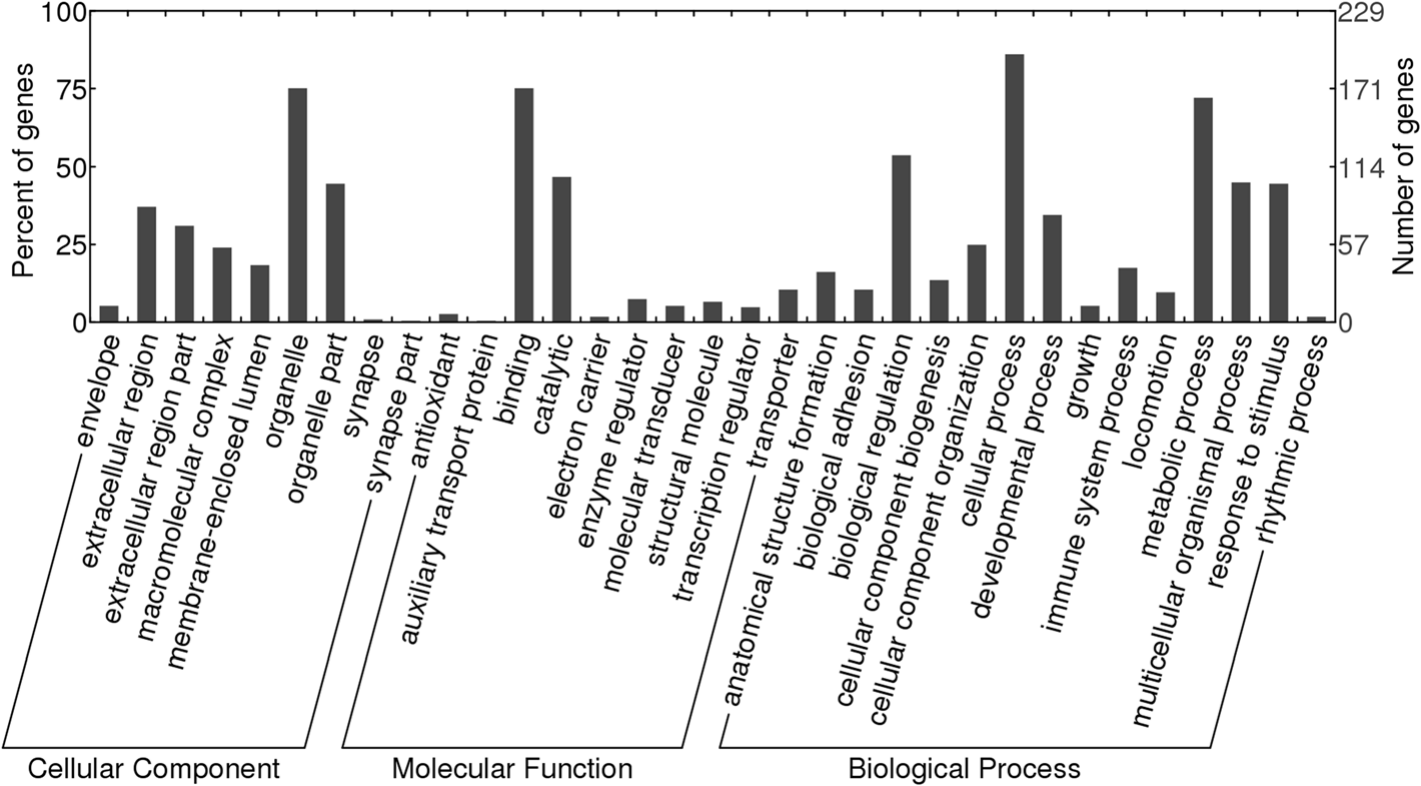
GO term analysis of regulated genes from the anterior intestinal mucosa of European seabass fed diets containing low FM diet (FM5) as compared to sea bass fed a high FM diet (FM20)

GO term analysis revealed that dietary FM reduction, from 20 to 5%, led to the regulation of a wide variety of genes involved in the metabolism, immunity, and tissue development of European seabass intestinal mucosa (Fig. 2).

**Fig. 3:**
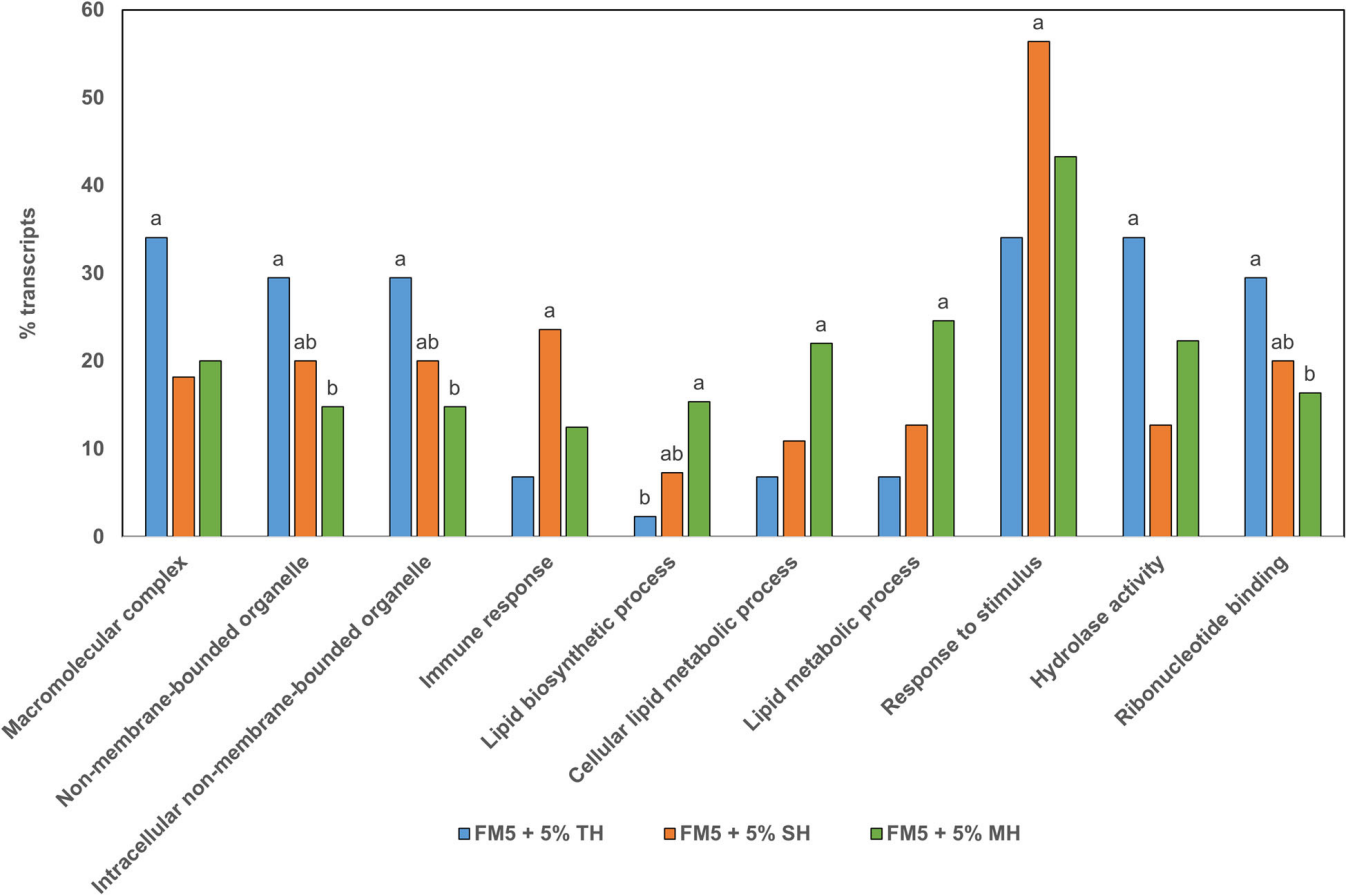
GO term analysis of regulated genes from the anterior intestinal mucosa of European seabass fed diets containing different types of hydrolysates as compared to sea bass fed a low FM diet (FM5). A Pearson Chi-Square test was performed separately for each GO term annotation to reveal statistical differences between diets. Histograms with the same superscript letter do not differ significantly (*P* > 0.05). FM5, 5% fish meal diet; TH, tilapia hydrolysate; SH, shrimp hydrolysate; MH, mixed hydrolysate

GO term analysis of the genes regulated by hydrolysate-supplemented diets is presented in Fig. 3. The specific hydrolysates, which were influenced by by-product origin and the hydrolysis process, differentially impacted gene regulation (*P* < 0.05). In particular, under the TH diet, the level of GO term annotation was higher for the following categories: macromolecular complex, non-membrane-bounded organelle, intracellular non-membrane-bounded organelle, hydrolase activity, and ribonucleotide binding. In contrast, under the SH diet, the differentially regulated genes belonged mostly to the immune response and response to stimulus. Finally, under the MH diet, lipid metabolism-related GO terms were over-represented.

Based on GO term annotations, 74 differentially expressed transcripts were linked to nutrition as compared to the FM5 diet: 15 transcripts related to digestion, 15 to carbohydrates, 7 to starvation, and 37 to carriers (Table 4). Treatments including tilapia hydrolysate (TH and MH diets) impacted the expression of digestive enzyme and carbohydrate metabolism genes more deeply than the FM20 diet, although SH inclusion in the FM5 diet modulated a limited number of genes (5). Under the FM20 diet, carrier genes were over-expressed, except for a few related to lipid transport (apolipoproteins). Diet formulations induced specific regulations with 12, 4, 4, and 32 transcripts differentially expressed by the FM20, SH, TH, and MH diets, respectively, as compared to FM5.

**Table 4:** Summary of differentially expressed genes related to nutrition

Classification/Transcript	Name (best blast)	Gene fold change as compared to the low fish meal diet (FM5)			
		FM20	FM5 + 5% TH	FM5 + 5% SH	FM5 + 5% MH
Digestion related					
TRINITY DN79635 c16 g28	2-phosphoxylose phosphatase 1	−1.85			
TRINITY DN74666 c1 g3	Aminopeptidase N	1.97			2.92
TRINITY DN74666 c0 g1	Aminopeptidase N				2.80
TRINITY DN76691 c3 g3	Chymotrypsin-C		2.30		
TRINITY DN66951 c0 g2	Dipeptidase 1				2.68
TRINITY DN75551 c3 g1	Dipeptidyl peptidase 4	1.68			1.96
TRINITY DN71008 c5 g1	Meprin A subunit alpha	1.54			
TRINITY DN72685 c5 g4	Meprin A subunit beta	1.90			2.07
TRINITY DN75683 c2 g1	Meprin A subunit beta	1.64	1.98		2.04
TRINITY DN6986 c0 g1	Pepsin A		3.11	2.7	4.79
TRINITY DN78404 c2 g1	Phospholipase B1, membrane-associated				3.61
TRINITY DN77910 c4 g1	Phospholipase B1, membrane-associated				4.33
TRINITY DN56338 c0 g1	Trypsinogen-like protein 3				−1.90
TRINITY DN74387 c3 g1	Xaa-Pro aminopeptidase 2	2.26			2.61
TRINITY DN75167 c0 g2	Xaa-Pro dipeptidase				1.72
Carbohydrate related					
TRINITY DN74193 c6 g1	2-oxoglutarate dehydrogenase, mitochondrial	1.82			
TRINITY DN73012 c2 g2	ADP-dependent glucokinase				−2.19
TRINITY DN77048 c0 g5	Beta-galactosidase				−2.18
TRINITY DN77546 c2 g14	Beta-hexosaminidase subunit beta				3.15
TRINITY DN76262 c5 g1	CMP-N-acetylneuraminate-beta-galactosamide-alpha-2,3-sialyltransferase 2	6.76		5.3	6.90
TRINITY DN76669 c2 g6	CMP-N-acetylneuraminate-beta-galactosamide-alpha-2,3-sialyltransferase 4	2.40			
TRINITY DN67442 c0 g3	Fructose-1,6-bisphosphatase 1		1.99		
TRINITY DN79451 c1 g1	Fructose-bisphosphate aldolase B		2.05		2.17
TRINITY DN77757 c0 g1	Glucose-6-phosphatase				2.48
TRINITY DN78836 c3 g3	Glucose-6-phosphatase		2.99		3.71
TRINITY DN77732 c1 g2	Lysosomal alpha-mannosidase				2.13
TRINITY DN75851 c2 g4	Maltase-glucoamylase, intestinal	2.10	2.31		3.03
TRINITY DN75571 c0 g1	Phosphoenolpyruvate carboxykinase, cytosolic [GTP]	1.70			2.34
TRINITY DN66475 c0 g1	UDP-glucose 4-epimerase	−2.69			−2.53
TRINITY DN75517 c0 g1	UDP-glucose 6-dehydrogenase				−4.48
Starvation related					
TRINITY DN71986 c0 g1	Angiopoietin-related protein 4	−1.71			−2.88
TRINITY DN65255 c0 g1	Collectrin				4.96
TRINITY DN69741 c0 g2	DEP domain-containing mTOR-interacting protein	2.10			1.87
TRINITY DN79325 c3 g4	Folliculin-interacting protein 1	1.69			
TRINITY DN53239 c0 g1	Neuropeptide Y				−3.68
TRINITY DN59469 c0 g1	Neuropeptide YY-A				−2.59
TRINITY DN76722 c3 g1	Serine/threonine-protein kinase ULK2	2.57			
Carrier					
TRINITY DN79318 c11 g9	14 kDa apolipoprotein	−1.64			−2.91
TRINITY DN79318 c11 g7	14 kDa apolipoprotein	−1.79			−2.92
TRINITY DN69453 c0 g1	Apolipoprotein A1/A4/E domain (pfam)	−3.09			−7.19
TRINITY DN71711 c0 g1	Apolipoprotein A-IV	−2.02			− 2.84
TRINITY DN70740 c0 g1	Apolipoprotein A-IV	−3.28		−2.1	−5.05
TRINITY DN79385 c0 g8	Apolipoprotein B-100		2.57		
TRINITY DN73752 c8 g3	Apolipoprotein C-I (ApoC-1) pfam				−3.87
TRINITY DN78962 c1 g23	Apolipoprotein C-II (pfam)				−2.29
TRINITY DN78962 c1 g36	Apolipoprotein C-II (pfam)	−1.66			−2.54
TRINITY DN78793 c2 g9	Apolipoprotein Eb				−4.81
TRINITY DN63420 c0 g2	Aquaporin FA-CHIP				2.00
TRINITY DN76968 c1 g2	Asc-type amino acid transporter 1	1.70			
TRINITY DN70677 c12 g4	ATP-binding cassette sub-family A member 1				1.98
TRINITY DN78213 c1 g1	Chloride anion exchanger				2.74
TRINITY DN68770 c0 g2	Chloride intracellular channel protein 4				2.01
TRINITY DN67686 c1 g1	Chloride intracellular channel protein 5				1.77
TRINITY DN69002 c1 g2	Large neutral amino acids transporter small subunit 4				1.96
TRINITY DN79534 c0 g1	MLN64 N-terminal domain homolog				2.39
TRINITY DN75016 c2 g2	Monocarboxylate transporter 12-B				1.81
TRINITY DN71474 c7 g3	Na(+)/H(+) exchange regulatory cofactor NHE-RF1				2.64
TRINITY DN74500 c2 g1	Na(+)/H(+) exchange regulatory cofactor NHE-RF3				1.94
TRINITY DN79418 c0 g1	Niemann-Pick C1-like protein 1				2.65
TRINITY DN76301 c3 g1	Niemann-Pick C1-like protein 1				1.85
TRINITY DN75948 c4 g4	Phospholipid-transporting ATPase IA	2.26			
TRINITY DN75214 c1 g9	Prolyl endopeptidase-like				1.85
TRINITY DN77239 c2 g2	Sodium/hydrogen exchanger 7				4.65
TRINITY DN67206 c1 g1	Sodium/myo-inositol cotransporter	−3.06			−3.55
TRINITY DN67731 c1 g1	Sodium/potassium-transporting ATPase subunit alpha-1		2.22		2.99
TRINITY DN79640 c8 g1	Sodium-dependent neutral amino acid transporter B(0)AT1	2.83		2.7	3.96
TRINITY DN71667 c2 g2	Sodium-dependent phosphate transport protein 2B			2.3	3.25
TRINITY DN69556 c1 g3	Solute carrier family 13 member 2	2.15			2.78
TRINITY DN60850 c0 g1	Solute carrier family 13 member 5	2.34		2.1	2.73
TRINITY DN68059 c1 g4	Solute carrier family 15 member 1		1.78		
TRINITY DN73228 c5 g1	Solute carrier family 2, facilitated glucose transporter member 2				2.56
TRINITY DN72783 c3 g1	Solute carrier family 22 member 6		−3.83		
TRINITY DN79648 c5 g9	Solute carrier family 40 member 1				2.60
TRINITY DN73224 c8 g2	Zinc transporter 1				2.54

Values correspond to fold changes between diets and the low fish meal control diet. Positive values indicate upregulation, and negative values down regulation. *FM5* 5% fish meal diet, *FM20* 20% fish meal diet, *TH* tilapia hydrolysate diet, *SH* shrimp hydrolysate diet, *MH* mixed hydrolysate diet

Table 5 presents regulated transcripts related to the cholesterol and long-chain fatty acid pathways. Lipid metabolism was modulated by the MH diet even more than by the FM20 diet. As compared to the FM5 diet, 15, 0, 3, and 28 transcripts were differentially expressed by the FM20, TH, SH and MH diets, respectively. The TH diet did not impact the expression of the transcripts related to the cholesterol and long-chain fatty acid pathways.

**Table 5:** Summary of differentially expressed genes related to cholesterol and long chain fatty acid pathways

Classification/Transcript		Gene fold change as compared to the low fish meal diet (FM5)			
		FM20	FM5 + 5% TH	FM5 + 5% SH	FM5 + 5% MH
Cholesterol related					
TRINITY DN71881 c0 g2	3-beta-hydroxysteroid-Delta(8),Delta(7)-isomerase				−3.00
TRINITY DN67458 c2 g3	Delta(14)-sterol reductase				−2.75
TRINITY DN76391 c4 g1	Farnesyl pyrophosphate synthase	−2.98		−2.7	−5.68
TRINITY DN71950 c2 g1	Hydroxymethylglutaryl-CoA synthase, cytoplasmic	−1.93			−3.19
TRINITY DN79342 c1 g2	Lanosterol 14-alpha demethylase	−1.85			−2.06
TRINITY DN75940 c1 g2	Lanosterol synthase	−2.65			−3.74
TRINITY DN76284 c0 g1	Lathosterol oxidase				−2.68
TRINITY DN72542 c3 g1	Neutral cholesterol ester hydrolase 1	2.61			
TRINITY DN66926 c0 g1	Squalene monooxygenase				−3.82
TRINITY DN78143 c3 g1	Squalene synthase				−2.64
TRINITY DN71672 c0 g3	Sterol-4-alpha-carboxylate 3-dehydrogenase, decarboxylating				−3.59
TRINITY DN75298 c0 g2	Transmembrane protein 97				−2.25
Fatty acid related					
TRINITY DN70866 c4 g9	2,4-dienoyl-CoA reductase, mitochondrial				−1.92
TRINITY DN78203 c0 g3	3-ketoacyl-CoA thiolase, mitochondrial				−2.37
TRINITY DN74534 c0 g3	Acetoacetyl-CoA synthetase				−4.95
TRINITY DN74255 c1 g3	Acetyl-CoA carboxylase				−2.11
TRINITY DN72189 c4 g3	Acyl-CoA desaturase	1.78			5.40
TRINITY DN70036 c1 g2	Alkylglycerol monooxygenase	1.99			
TRINITY DN77637 c3 g4	ATP-binding cassette sub-family D member 2				−3.41
TRINITY DN74635 c4 g2	Elongation of very long chain fatty acids protein 4				−2.71
TRINITY DN73852 c1 g2	Elongation of very long chain fatty acids protein 6				−3.96
TRINITY DN79046 c1 g4	Fatty acid hydroxylase domain-containing protein 2				1.94
TRINITY DN79053 c1 g4	Fatty acid synthase				−3.33
TRINITY DN79341 c1 g1	Fatty acid-binding protein, brain	4.26			
TRINITY DN56507 c0 g1	Fatty acid-binding protein, intestinal				−1.88
TRINITY DN79266 c1 g10	Fatty acid-binding protein, liver-type				−2.17
TRINITY DN78522 c0 g2	Long-chain-fatty-acid--CoA ligase 4	−1.89			
TRINITY DN75220 c0 g3	Methylsterol monooxygenase 1	−2.26			−2.31
TRINITY DN78695 c0 g7	Perilipin-2	−3.04			
TRINITY DN78695 c0 g2	Perilipin-2	−3.15		−2.3	
TRINITY DN72627 c0 g1	Phosphatidate phosphatase LPIN1				−4.12
TRINITY DN72627 c1 g3	Phosphatidate phosphatase LPIN2	2.00			
TRINITY DN71726 c1 g1	Sodium-dependent lysophosphatidylcholine symporter 1-B				−3.38
TRINITY DN72471 c1 g2	Trifunctional enzyme subunit beta, mitochondrial				−2.18
TRINITY DN67880 c1 g2	Very long-chain acyl-CoA synthetase			2.1	3.01
TRINITY DN71905 c5 g2	Very long-chain acyl-CoA synthetase	1.93			
TRINITY DN76293 c0 g2	Very long-chain acyl-CoA synthetase	1.87			

Values correspond to fold changes between diets and the low fish meal control diet. Positive values indicate upregulation, and negative values down regulation. *FM5* 5% fish meal diet, *FM20* 20% fish meal diet, *TH* tilapia hydrolysate diet, *SH* shrimp hydrolysate diet, *MH* mixed hydrolysate diet

Transcripts related to fish immunity and/or the stress response are presented in Table 6. As compared to the FM5 diet, 20, 4, 12, and 36 transcripts were differentially expressed by the FM20, TH, SH and, MH diets, respectively. In contrast to the FM20 diet, all the diets containing hydrolysate down-regulated the expression of transcripts related to interferon. The diets containing one kind of hydrolysate (TH or SH) did not impact the regulation of histo-compatibility antigen-related genes, and only slightly impacted the serum complement- and cellular damage-related genes. Lectin-related gene expression was not affected by dietary TH inclusion.

**Table 6:** Summary of differentially expressed genes related to fish health status

Classification/Transcript	Name (best blast)	Gene fold change as compared to the low fish meal diet (FM5)			
		FM20	FM5 + 5% TH	FM5 + 5% SH	FM5 + 5% MH
Interferon related					
TRINITY DN69994 c1 g3	Gamma-interferon-inducible lysosomal thiol reductase				2.39
TRINITY DN67652 c1 g1	Interferon alpha-inducible protein 27-like protein 2A			−2.2	−2.22
TRINITY DN78765 c2 g3	Interferon stimulated gene 15			−3.4	
TRINITY DN78866 c1 g8	Interferon-induced GTP-binding protein Mx		−2.72		−2.56
TRINITY DN78866 c1 g9	Interferon-induced GTP-binding protein Mx		−3.37	−2.7	−2.41
TRINITY DN78532 c1 g14	Interferon-inducible protein 56			−2.3	−2.23
Histocompatibility antigen related					
TRINITY DN79419 c7 g6	Class I histocompatibility antigen, B alpha chain	1.95			2.98
TRINITY DN79651 c13 g9	H^−2^ class I histocompatibility antigen, K-K alpha chain				3.85
TRINITY DN78477 c2 g7	H-2 class II histocompatibility antigen gamma chain	1.95			2.74
TRINITY DN72971 c0 g4	Rano class II histocompatibility antigen, A beta chain	2.20			2.45
TRINITY DN72971 c0 g3	Rano class II histocompatibility antigen, A beta chain				3.32
Lectin related					
TRINITY DN69402 c2 g1	Fucolectin	4.15		3.9	5.35
TRINITY DN69402 c2 g2	Fucolectin	6.15		2.9	7.67
TRINITY DN65160 c0 g3	Fucolectin-1	2.46			3.39
TRINITY DN65160 c0 g4	Fucolectin-1			4.2	7.27
TRINITY DN78379 c3 g2	Fucolectin-1				5.03
TRINITY DN78379 c3 g7	Fucolectin-1	2.54			2.38
TRINITY DN69906 c1 g1	Nattectin				3.37
Pentraxin related					
TRINITY DN77743 c1 g13	C-reactive protein			2.9	
TRINITY DN77743 c1 g7	C-reactive protein	3.73	3.25	2.5	4.47
TRINITY DN14972 c0 g1	Serum amyloid P-component	5.12		2.8	5.87
Serum complement related					
TRINITY DN60272 c0 g1	C1q domain (pfam)	1.53			
TRINITY DN60272 c0 g1	C1q domain (pfam)	1.53			
TRINITY DN78829 c1 g1	C1q domain (pfam)	3.53			4.69
TRINITY DN77811 c0 g3	C1q domain (pfam)				2.91
TRINITY DN73741 c7 g14	C1q-like 23 kDa protein		6.85		
TRINITY DN67406 c0 g1	C1q-like 23 kDa protein	7.94			10.81
TRINITY DN79561 c2 g21	C1q-like 23 kDa protein	6.25			10.35
TNF-α related					
TRINITY DN59663 c0 g1	Tumor necrosis factor alpha-induced protein 8-like protein 3				2.31
TRINITY DN75139 c4 g2	Tumor necrosis factor ligand superfamily member 10	2.20		2.3	3.09
TRINITY DN69734 c2 g1	Tumor necrosis factor receptor superfamily member 11A				2.82
Cellular stress related					
TRINITY DN71217 c2 g2	Glutathione peroxidase 2				−2.58
TRINITY DN79626 c5 g2	Phospholipid hydroperoxide glutathione peroxidase, mitochondrial	−2.21			−3.81
TRINITY DN79626 c5 g6	Phospholipid hydroperoxide glutathione peroxidase, mitochondrial				−3.27
TRINITY DN79626 c5 g1	Phospholipid hydroperoxide glutathione peroxidase, mitochondrial	−2.56			−4.22
TRINITY DN76439 c4 g1	Gamma-glutamyltranspeptidase 1	2.72			4.40
TRINITY DN63786 c0 g2	Sestrin-1				2.12
TRINITY DN77226 c0 g3	Selenocysteine lyase				−3.08
TRINITY DN68233 c1 g1	DNA damage-inducible transcript 4-like protein	−6.45		−3.2	−3.41
TRINITY DN77069 c1 g2	DNA repair protein complementing XP-A cells homolog	−2.82			−2.64
TRINITY DN76110 c0 g10	Tyrosyl-DNA phosphodiesterase 2				−2.57

Values correspond to fold changes between diets and the low fish meal control diet. Positive values indicate upregulation, and negative values down regulation. *FM5* 5% fish meal diet, *FM20* 20% fish meal diet, *TH* tilapia hydrolysate diet, *SH* shrimp hydrolysate diet, *MH* mixed hydrolysate diet

### Impacted metabolic pathways

We conducted various analyses to study the metabolic pathways impacted by dietary hydrolysates. Firstly, we performed a string analysis (https://string-db.org/cgi/input.pl?) to reveal relationships between regulated genes and a defined enrichment (Additional file 4). Regarding the TH diet, 11 genes, distributed into 3 groups, were interconnected, but no enrichment was observed. In the case of the SH diet, 11 regulated genes formed 2 groups, but without inducing overexpression of any specific metabolic pathway. Six regulated genes were common to the TH and SH diets (*cebpd, cebpb, rsad2, herc6, mx1 and cmpk2*). The *g6pc, aldob, eif2ak4, ace, apob* and *hba1, scn2a, junb, fdp5, dhx58* genes were connected to the TH and SH diets, respectively. Under the MH diet, many more regulated genes were connected (87), with the ACLY gene at the center of the gene network. The enrichment scores calculated for biological processes, molecular functions, cellular component and KEGG pathways are shown in Table 7. The main enrichments for biological processes were the following: small molecule metabolic process, cellular lipid metabolic process, lipid metabolic process, organophosphate metabolic process, single-organism catabolic process; cellular components were cytosol, cytoplasmic part, membrane-bounded vesicle, extracellular exosome, and extracellular region part; whereas molecular functions were catalytic activity, ethanolamine kinase activity, oxidoreductase activity, cofactor binding, coenzyme binding, and KEGG metabolic pathways were steroid biosynthesis, fat digestion and absorption, glycerophospholipid metabolism, and glycolysis/gluconeogenesis.

**Table 7:** Functional enrichments related to the mixed hydrolysate diet (FM5 + 5% MH) as compared to the low FM diet (FM5)

Pathway ID	Pathway description	Count in gene set	False discovery rate
Biological Process (GO)			
GO:0044281	small molecule metabolic process	47	1.29E-10
GO:0044255	cellular lipid metabolic process	27	1.35E-08
GO:0006629	lipid metabolic process	30	2.85E-08
GO:0019637	organophosphate metabolic process	25	1.72E-07
GO:0044712	single-organism catabolic process	26	2.12E-07
Molecular Function (GO)			
GO:0003824	catalytic activity	71	4.72E-08
GO:0004305	ethanolamine kinase activity	4	4.71E-06
GO:0016491	oxidoreductase activity	20	2.75E-05
GO:0048037	cofactor binding	13	2.75E-05
GO:0050662	coenzyme binding	11	4.17E-05
Cellular Component (GO)			
GO:0005829	cytosol	49	5.48E-06
GO:0044444	cytoplasmic part	80	7.17E-06
GO:0031988	membrane-bounded vesicle	49	1.27E-05
GO:0070062	extracellular exosome	43	1.27E-05
GO:0044421	extracellular region part	50	2.76E-05
KEGG Pathways			
1100	Metabolic pathways	32	4.73E-09
100	Steroid biosynthesis	4	0.000973
4975	Fat digestion and absorption	5	0.000973
564	Glycerophospholipid metabolism	6	0.00308
10	Glycolysis/Gluconeogenesis	4	0.0473

A KEGG analysis based on KEGG orthology annotation showed that the main impacted pathways differed depending on the origin of the dietary hydrolysate (Table 8). In particular, 9, 9, and 55 differentially regulated genes participated to metabolic pathways (KO: 01100) in response to the TH, SH, and MH diets, respectively. To be more precise, the metabolic pathways impacted by the TH diet mainly influenced carbohydrate metabolism, whereas the SH diet impacted the nucleotide metabolism and terpenoid backbone biosynthesis. In addition to these metabolic pathways, defense and immunity pathways were also affected by hydrolysate inclusion to the diet. The complete list of impacted pathways and corresponding genes is available in Additional file 5.

**Table 8:** KEGG analysis of the top 5 pathways regulated by hydrolysate-supplemented diets as compared to the low FM diet (FM5)

TH diet	Nb. of regulated genes
01100 Metabolic pathways	9
04974 Protein digestion and absorption	6
03010 Ribosome	3
04972 Pancreatic secretion	3
01120 Microbial metabolism in diverse environments	
SH diet	Nb. of regulated genes
01100 Metabolic pathways	9
04210 Apoptosis	5
04217 Necroptosis	4
05164 Influenza A	4
01110 Biosynthesis of secondary metabolites	3
MH diet	Nb. of regulated genes
01100 Metabolic pathways	55
01110 Biosynthesis of secondary metabolites	28
01130 Biosynthesis of antibiotics	19
00100 Steroid biosynthesis	9
05166 HTLV-I infection	9

*TH* tilapia hydrolysate, *SH* shrimp hydrolysate, *MH* mixed hydrolysate

We also investigated the pathways regulated by dietary treatments using Ingenuity Pathway Analysis Software. Results are summarized in Table 9, focusing on the top canonical pathways as well as on molecular and cellular functions. The main metabolic pathways impacted by dietary hydrolysates were the ones related to the carbohydrate and lipid metabolisms. Complete results are available in Additional file 6.

**Table 9:** Ingenuity Pathway Analysis: main pathways impacted by hydrolysate-supplemented diets

TH diet versus FM5 diet	SH diet versus FM5 diet	MH diet versus FM5 diet
Top Canonical Pathways (Name/p-value/Overlap)	Top Canonical Pathways (Name/p-value/Overlap)	Top Canonical Pathways (Name/*p*-value/Overlap)
Glycolysis I/1.23E-03/8%	Role of Lipids/Lipid Rafts in the Pathogenesis of Influenza/9.11E-04/9.1%	Superpathway of Cholesterol Biosynthesis/2.13E-17/42.9%
Gluconeogenesis I/1.23E-03/8%	Glutamine Biosynthesis I/2.04E-03/100%	Cholesterol Biosynthesis I/6.27E-16/69.2%
Glutamine Biosynthesis I/2.08E-03/100%	Xanthine and Xanthosine Salvage/2.04E-03/100%	Cholesterol Biosynthesis II (via 24.25-dihydrolanosterol) / 6.27E-16/69.2%
FXR/RXR Activation/2.30E-03/2.4%	Type II Diabetes Mellitus Signalling/2.20E-03/2.4%	Cholesterol Biosynthesis III (via Desmosterol)/6.27E-/ 69.2%
IL-17A Signalling in Fibroblasts/2.41E-03/5.7%	IL-17A Signalling in Fibroblasts/2.31E-03/5.7%	Zymosterol Biosynthesis/1.48E-07/66.7%
Molecular and Cellular Functions (Name/p-value/#Molecules)	Molecular and Cellular Functions (Name/*p*-value/#Molecules)	Molecular and Cellular Functions (Name/*p*-value/#Molecules)
Cell Morphology/2.27E-02 - 1.27E-05/9	Cell Death and Survival/1.73E-02 - 1.81E-05/14	Lipid Metabolism/6.58E-03 - 2.66E-14/79
Carbohydrate Metabolism/2.67E-02 - 6.33E-05/9	Cell-To-Cell Signalling and Interaction/1.62E-02 - 2.68E-05/10	Small Molecule Biochemistry/6.58E-03 - 2.66E-14/106
Small Molecule Biochemistry/2.75E-02 - 6.33E-05/20	Cellular Development 1.72E-02 -/3.65E-05/15	Vitamin and Mineral Metabolism/6.25E-03 - 1.96E-11/30
Amino Acid Metabolism/2.67E-02 - 2.76E-04/4	Cellular Function and Maintenance/1.73E-02 - 3.65E-05/14	Molecular Transport/6.25E-03 - 3.02E-10/74
Post-Translational Modification/1.24E-02 - 2.76E-04/2	Cellular Growth and Proliferation/1.72E-02 - 3.65E-05/12	Carbohydrate Metabolism/6.25E-03 - 1.60E-08/43

*TH* tilapia hydrolysate, *SH* shrimp hydrolysate, *MH* mixed hydrolysate, *FM5* low fish meal diet

## Discussion

### Impact of hydrolysate inclusion in a low FM diet on European seabass growth performances and histological organization of the intestine mucosa

Our results showed that a 15% decrease of dietary FM negatively affected the growth performances of European seabass as compared to the 20% FM control diet. These findings are consistent with other studies about other carnivorous fish species [12, 67–69]. However, dietary inclusion of 5% of protein hydrolysate restored growth performances to the same level as the FM20 control diet. These positive effects of dietary hydrolysates are in agreement with other studies about fish fed diets incorporating such ingredients [29, 32, 38].

Feed intake was not modified by diets as fish were fed in excess to guarantee a high consumption. Feed conversion ratio improvement of fish fed the FM20 diet compared to the FM5 diet could be the result of a better nutritional balance of the diet combined to a better feed consumption. Dietary inclusion of hydrolysate allowed to recover a good feed intake as well as to improve enough feed efficiency to reach fish and feed performance very closed to the positive control. The high degree of protein hydrolysis of our hydrolysates [37, 40], resulting in enrichment of the feed with highly palatable and digestible low-molecular-weight peptides and free amino acids, could explain why growth performances were improved. However, fish growth performances should not be the single criterion to evaluate the performance of a new aquafeed formula. It is also necessary to study the response of fish metabolism to ensure that modifications in the diet will not induce significant metabolic disturbances that could affect fish resistance to their environment. Maintaining the integrity of fish intestines is of critical concern when considering the performance of aquaculture feeds. Many studies have shown that enteritis events can occur when carnivorous fish are fed feeds formulated with high levels of plant-based proteins [17, 70–74]. A direct consequence of enteritis is deterioration of feed utilization and of the fish health status. The intestine is involved not only in digestion and feed absorption, but also in water and electrolyte balance, nutrient sensing, and immunity. This functional diversity is currently being elucidated in fish, and different histological and molecular approaches are helping to understand the many vital functions conducted along the gastrointestinal tract [46]. The general organization and morphometric parameters of the intestine, represented by villi size and the number of goblet cells, are good indicators of the health and condition of the fish intestinal mucosa: an increase of intestinal villi size reflects an improvement of the exchange surface, of the activity of the brush border enzymes and of the nutrient transport systems, with positive effects on digestion and absorption [75]. In addition, in rainbow trout (*O. mykiss*) goblet cells regulate proteins or peptides as well as ion and fluid transport, and also provide an effective immune barrier against potentially pathogenic gut bacteria [47, 76]. Furthermore, enhanced mucin production by increased goblet cell populations physically displaces potentially pathogenic organisms, a more diverse microbiota leads to a thickening of the mucus layer, and this improves gut microniches inhabited by these beneficial bacteria [77]. In the present study, we demonstrate that European seabass fed a diet containing low fish meal levels (FM5) had an altered intestinal mucosa. These results are consistent with other studies that showed a decrease in villi height [39, 72, 78–80] and/or in the number of goblet cells [38, 39] in fish fed the low FM diet. Incorporation of hydrolysates at a relatively low inclusion rate (5%) in the low FM formula (FM5) improved the histological organization of the intestinal mucosa, with villi heights close to those observed in the control group (FM20). Moreover, the origin of the raw material used to manufacture the hydrolysates, as well as their hydrolysis level, seemed to be determining parameters for enhancing the morphological development of the intestine. The different specifications of the protein hydrolysates led to different responses of villi height, with a higher benefit from dietary SH than TH, although MH performance lay in-between. However, it is still unclear whether this improved response was related to a dietary effect of SH on the intestinal mucosa due to i) the higher intake of free amino acids, or ii) a protective effect of the intestinal epithelium due to the presence of bioactive peptides in the hydrolysate, or iii) a modulation of the intestinal microbiota and/or of antimicrobial peptides. These hypotheses are not mutually exclusive. Although the mode of action of hydrolysates remains to be elucidated, this study confirms that they are beneficial for the fish intestine, as already observed in olive flounder [39]. It also confirms the potential of hydrolysates for replacing FM in aquafeeds for carnivorous species.

### Impact of hydrolysate inclusion in a low FM diet on intestine gene expression

The intestine is a complex organ because many metabolic interactions take place inside it [49]. We analyzed the differentially expressed genes from the intestinal mucosa of European seabass fed different diets. Low FM (FM5 diet) caused metabolic disturbances, and certain metabolic pathways were modulated in link with dietary inclusion of protein hydrolysates of different origins. Dietary FM reduction (FM5 diet) impaired several intestinal functions, i.e. nutrient transport, immune defense, gut morphogenesis (Fig. 2). These transcriptomic results are consistent with the lower performances recorded in sea bass fed the FM5 diet throughout the zootechnical trial.

The different transcriptomic data analyses evidenced a few discrepancies in sea bass responses depending on dietary hydrolysate origin (Tables 8 and 9). The comparative analysis of the two SH and TH protein hydrolysates confirmed that the sea bass intestinal transcriptomic response seemed to be closely related to the biochemical properties of each hydrolysate. Thus, based on GO term analysis, a higher proportion of genes (34.1% vs. 12.7%) linked to hydrolases was differentially expressed in sea bass fed the TH diet as compared to the SH diet (Fig. 3). Interestingly, the regulated enzymes were not only implied in peptide/protein degradation, as expected from dietary protein hydrolysate inclusion, but also in carbohydrate degradation (malatase-gluco-amylase, aldolase B, glucose-6-phosphatase), and probably stimulate starch degradation in the PBM fraction of diets (Table 4). These differences in gene expression of intestinal hydrolases might be explained by the different peptide compositions of tilapia and shrimp hydrolysates. TH is mainly composed of higher-molecular-weight peptides than SH, and also exhibits higher peptide diversity than SH [40]. These higher-molecular-weight peptides could require additional hydrolysis by enterocyte enzymes, resulting in greater mRNA synthesis from genes related to protein digestion and absorption. On the contrary, GO terms of differentially expressed genes involved in the immune response (6.8% vs. 23.6%) and in the response to stimuli (34% vs. 56.4%) were less in sea bass fed the TH diet than in sea bass fed the SH diet (Fig. 3). In particular, the SH diet induced the expression of lectin-related genes (Table 6), which protect the intestinal mucosa from pathogenic bacterial invasion [81]. These findings support the immunostimulating effect of SH. SH is mainly composed of low-molecular-weight peptides derived from the enzymatic cleavage of haemocyanin [37], which has immunostimulatory properties used in the treatment of cancers such as melanomas or in bioadjuvants for vaccines [82–84]. In addition, peptides derived from haemocyanin degradation are also known to exhibit other bioactive properties such as responses to stressors, pathogens (bacteria and parasites) and antibacterial agents [85]. Marine protein hydrolysates have been reported to have in vitro antibacterial [37, 40], antioxidant and/or immunomodulatory [86] functional properties. Our results seem to confirm immuno-stimulatory effects (interferon, histocompatibility antigen, lectin, pentraxin or serum complement system expression) at a transcriptomic level, as already observed in several carnivorous fish fed diets containing the same hydrolysates [32, 33, 38, 39, 87]. Moreover, hydrolysates down-regulated the expression of interferon-related genes. In fish, genes linked to interferon are expressed during viral infection [81], and in vertebrates, interferons are also involved in intestinal homeostasis after infection [88]. These transcriptomic results confirm that dietary hydrolysate supplementation has beneficial effects on the intestinal mucosa.

String analysis did not evidence any GO term or metabolic enrichment in diets containing hydrolysates of a specific origin (shrimp or tilapia). This result is surprising given the marked difference in growth responses between sea bass fed the FM5 diet and sea bass fed diets containing hydrolysates, as well as the positive response of intestine histological organization in sea bass fed the SH diet. For the SH diet, this may be due to the fact that sea bass fed the FM5 diet did not show any histological signs of severe enteritis or inflammation of the intestinal mucosa; besides, there was no change at a molecular level. Enteritis induces the regulation of a large number of genes (apoptosis, proinflammatory, oxidative stress, endocytosis, and cell migration) as already shown in a study on the replacement of FM by soybean meal in Atlantic salmon [48]. In our study, the physiological state of the sea bass may not have been sufficiently altered to induce a transcriptomic response taking into account our filtering treatment (absolute fold change > 1.4 and *P-adj* < 0.2). Hence, the low number of regulated genes did not allow us to highlight any group of genes to explain the phenotypic differences observed between dietary treatments at a transcriptomic level.

On the contrary, in sea bass fed the diet containing the mixed hydrolysate (MH diet), the much larger number of differentially regulated genes (270 as compared to the FM5 diet) made it possible to carry out an enrichment analysis with String both in GO terms and in metabolic pathways. The analysis showed that the lipid metabolism was strongly impacted, and acetyl-CoA appeared to play a central role in this process. Interestingly, the MH diet decreased cholesterol and long-chain fatty acid metabolism (Table 5). Genes involved in cholesterol and long-chain fatty acid metabolism were overexpressed in Atlantic salmon fed protein diets containing a high level of soybean meal [48]. On the contrary, these two genes families were down-regulated in European seabass fed the MH diet. Moreover, many genes coding for apolipoprotein, which is involved in lipid transport, were down-regulated in sea bass fed with the mixed hydrolysate. These results provide evidence for a significant effect of the mixed hydrolysate diet on lipid metabolism at the transcriptomic level. Such an effect would deserve to be deeply investigated to understand the phenotypic consequences (lipid utilization and storage) of such gene responses to dietary protein hydrolysate. It is worth noting that modulation of lipid metabolism by dietary protein hydrolysates has already been observed in turbot and mice, with a beneficial effect on the reduction of visceral lipid accumulation [35, 89–91].

The MH diet also promoted European seabass health status by regulating the same immuno-stimulatory related genes as the TH and SH diets, but also by specifically modulating the expression of specific genes, such as H^− 2^ class I and class II histocompatibility antigen genes, which were over-expressed (Table 6). Moreover, the genes involved in the response to cellular damage (glutathione peroxidase, DNA damage repair) were under-expressed, which suggests an improvement of intestinal health and condition. Król et al. demonstrated that deregulation of the intestine (enteritis) induced the overexpression of genes linked to the oxidative defenses of epithelial cells [48]. A possible explanation for this larger number of differentially regulated genes may be that the combined hydrolysates provided a wider variety of functional peptides, an important characteristic linked to the performance of dietary protein hydrolysates [34].

Nevertheless, a focus could be performed further by qPCR in order to target the major actors of the main metabolic pathways highlighted by this study.

### Restoring the intestinal transcriptomic profile of the high FM diet with a low FM diet supplemented with protein hydrolysates

Hydrolysate diets induced a similar pattern of gene expression as the control diet (FM20), regardless of the included hydrolysate. Compared to the FM20 diet, the number of differentially regulated genes was very low for all hydrolysate diets, with 6, 8, and 6 genes for the TH, SH, and MH diets, respectively (Additional file 3). Once again, regulated genes were specific to hydrolysate origin, so that no gene was regulated by all three TH, SH and MH diets as compared to the FM20 group. Another mechanism involved in the differences in gene regulation between hydrolysate diets may be related to the glycosylation types in the resulting peptide fragments. The method used to generate these hydrolysates may also influence the degree of conservation of the glycosidic residues. Immune stimulation was more prominent in sea bass fed with SH; in parallel, glycosylation patterns conserved among plants and invertebrates have been found to be immunogenic in vertebrates [92]. Further work in the area of protein glycosylation may also be profitable and provide insights into this hypothesis. Although it is difficult to conclude about the relationship between regulated genes and the functional benefits of the TH diet, our results show that the SH diet down-regulated members of the GTPase IMAP family (members 6, 7 and 8) related to immunity. This highlights again the immunomodulatory effect of SH. Regarding the MH diet, Acyl-CoA desaturase and UDP-glucose 6-dehydrogenase were respectively over- and under-expressed as compared to the FM20 diet. Therefore the lipid and carbohydrate pathways were modulated by this dietary treatment. Achieving the performance of a diet rich in FM during grow-out is a key point when dealing with low FM diets and alternative ingredients. In this study, the incorporation of 5% of hydrolysate compensated for the metabolic disturbances associated with the replacement of 15% of FM by a mixture of plant proteins, as observed in European seabass fed the FM5 diet. The intestinal transcriptomic response confirmed the results obtained at the zootechnical and gut histological levels, i.e. protein hydrolysates of aquaculture origin are valuable candidates to support FM replacement without deteriorating the zootechnical and functional performances of aquafeeds.

## Conclusion

Within the global context of FM replacement in aquaculture feed formulation, evaluating the performance of new raw materials has become a crucial economic stake for aquafeed manufacturers. Although assessing the zootechnical performances of new formulations remains indispensable, the development of new protocols and tools for molecular and biochemical investigations can provide a more comprehensive view of the response of animals to novel diets. We combined zootechnical and transcriptomic approaches to evaluate the performances of protein hydrolysates as surrogates of dietary FM in European seabass. The reduction of the dietary FM level from 20 to 5% significantly impaired growth performances, intestinal histological organization, and induced significant changes in the transcriptomic profile of the intestine. By incorporating protein hydrolysate into the low FM diet (FM5) restored European seabass performances to a similar high level to those recorded with the high FM diet (FM20). In addition, this study demonstrates that the raw material used to manufacture protein hydrolysate is an important determinant in the transcriptomic response of fish.

Although the hydrolysates promoted the same zootechnical performances, the transcriptomic response of the intestine showed that genes and metabolic pathways were modulated in a hydrolysate-dependent manner. Moreover, including a combination of tilapia and shrimp hydrolysates into European seabass feed regulated a higher number of genes than the independent hydrolysates. It would be interesting to assess hydrolysate performances in fish grown under more challenging conditions (high density, low oxygen, low/high temperature,…) to check how these differences in transcriptomic profiles are translated into phenotypic responses. These results offer an interesting scenario for the formulation of new high-performance feeds with a low level of FM inclusion. The analysis of the transcriptomic responses of liver and kidney sampled in the present study will allow us to complete this work and to better understand how metabolic pathways are modulated by the replacement of FM by protein hydrolysates in European seabass.

## Additional files

Additional file 1: Diet formulation and proximal composition. FM5, 5% fish meal diet; FM20, 20% fish meal diet; TH, tilapia hydrolysate diet; SH, shrimp hydrolysate diet; MH, mixed hydrolysate diet. (XLSX 10 kb)

Additional file 2: Global list and fold changes of differentially expressed genes versus FM5. Values correspond to fold changes between diets and the low fish meal control diet. Positive values indicate upregulation, and negative values down regulation. FM5, 5% fish meal diet; FM20, 20% fish meal diet; TH, tilapia hydrolysate diet; SH, shrimp hydrolysate diet; MH, mixed hydrolysate diet. Values with “*” correspond to fold change means of isoforms. If isoforms were at the same time up and down regulated each values appear. (XLSX 37 kb)

Additional file 3: Global list and fold changes of differentially expressed genes versus FM20. Values correspond to fold changes between hydrolysate diets and the high fish meal control diet. Positive values indicate upregulation, and negative values down regulation. (XLSX 10 kb)

Additional file 4: Relationship between regulated genes by hydrolysate diets. Differentially expressed gene compared to low fish meal diet have been tested against *Homo sapiens* background. Only connected genes are presented. A. relation between the regulated genes by shrimp hydrolysate diet. B. relation between the regulated genes by tilapia hydrolysate diet. C. relation between the regulated genes by mixed hydrolysate diet. (TIF 2612 kb)

Additional file 5: KEGG analysis: list of genes differentially expressed by hydrolysate and high fish meal diets compared to low fish meal diet. (XLSX 63 kb)

Additional file 6: IPA analysis: list of main pathways impacted by hydrolysate and high fish meal diets compared to low fish meal diet. (XLSX 57 kb)
