# Supplementary figures and images for: Dietary aquaculture by-product hydrolysates: impact on the transcriptomic response of the intestinal mucosa of European seabass (Dicentrarchus labrax) fed low fish meal diets
Source: BMC Genomics. 2018 May 24;19:396. doi: 10.1186/s12864-018-4780-0 (PMC5968468; doi:10.1186/s12864-018-4780-0)

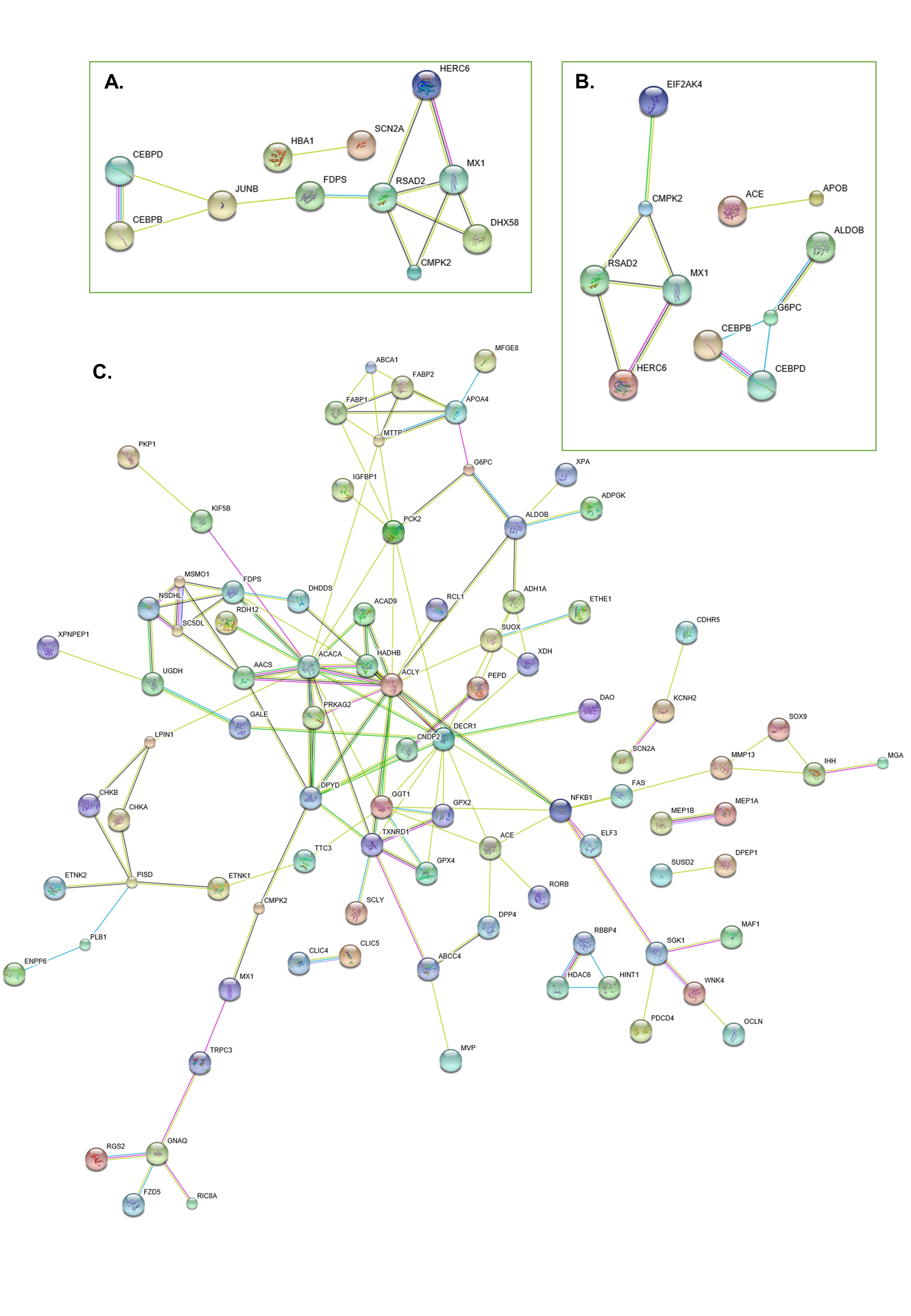

Supplement: Supplementary file 4 — Relationship between regulated genes by hydrolysate diets. Differentially expressed gene compared to low fish meal diet have been tested against Homo sapiens background. Only connected genes are presented. A. relation between the regulated genes by shrimp hydrolysate diet. B. relation between the regulated genes by tilapia hydrolysate diet. C. relation between the regulated genes by mixed hydrolysate diet. (TIF 2612 kb) [file 12864_2018_4780_MOESM4_ESM.tif]
